# Treatment for female patients with eating disorders in the largest medical prison in Japan

**DOI:** 10.1186/s13030-015-0040-6

**Published:** 2015-05-13

**Authors:** Tomokuni Asami, Maya Yanase, Toshiaki Nomura, Yoshiro Okubo

**Affiliations:** 1 Department of Psychiatry, Nippon Medical School, 1-1-5 Sendagi, Bunkyo-ku, Tokyo 113-8602 Japan; 2 Department of Psychiatry, Graduate School of Medicine, Kyoto University, 54 Shogoin-Kawaharacho, Sakyo-ku, Kyoto 606-8507 Japan; 3 Department of Medical Psychology, Nippon Medical School, 1-7-1, Kyonancho, Musashino, Tokyo 180-0023 Japan

**Keywords:** Anorexia nervosa, Eating disorder, Prison, Crime, Drug-offense, Correctional facility, Shoplifting, Excessive exercise, Antisocial behavior

## Abstract

The number of offenders with eating disorders in women’s prisons in Japan has grown annually over the last 15 years. Women’s prisons have experienced significant difficulties in the management of patients with eating disorders who have body-critical complications arising from low body weight, in addition to behavioral problems. Patients in Japan’s 185 correctional facilities who display high refractoriness or who present a physical risk are transferred to the Hachioji medical prison, a national specialty hospital operated by the Ministry of Justice. The medical prison must manage any psychosomatic problems necessary for the safety of inmates regardless of a patient’s wishes. The most common conviction resulting in imprisonment of women with eating disorders was shoplifting (*n* = 44; 63%), with the second most common being drug-offenses (*n* = 17; 24%). While shoplifting is of concern in relation to eating disorders, a causal relationship remains unclear. Most patients in the shoplifting group did not have histories of antisocial and/or impulsive behaviors such as drug abuse, sexual deviation, self-injury, or other criminal activity. Instead, shoplifting appears to be an obsessive-compulsive behavior deeply rooted in the psychopathology of severe eating disorder patients. Patients in this group tended to have histories of relatively high education and steady employment, although most also had histories of prolonged eating disorders and unstable treatment. Although adherence to treatment was poor among patients with eating disorders in the medical prison, body weight and behavioral problems improved following treatment in the special compulsory environment, without severe sequelae or patient death. The Ministry of Justice recently established another specialized ward for the care for female patients with eating disorders. If greater emphasis is placed on early-stage, protective, medical treatment, the number of patients with eating disorders in prisons may decrease. Further research is required to investigate the relationship between shoplifting and eating disorders.

## Introduction

The association between shoplifting and eating disorders has been a topic of discussion since the 1980s [1-6]. Shoplifting has been considered an impulsive behavior that accompanies eating disorders, similar to substance abuse, suicide attempts, self-injurious behavior, and sexual deviance [7-12], particularly in relation to multi-impulsive bulimia nervosa [13-17]. However, there is a paucity of research that focuses solely on patients with eating disorders who repeatedly shoplift or are actually incarcerated [18-22]. As shoplifting by patients with eating disorders has been identified as a significant problem in Japan [22-24], the present study reviewed the circumstances of incarcerated Japanese patients with eating disorders incarcerated.

**Figure 1:**
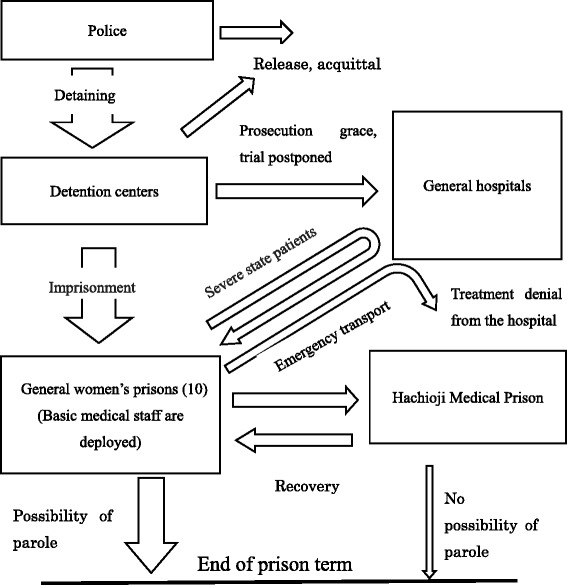
Transfer of female inmates with mental or physical diseases within Japanese correctional facilities (Reorganization of the figure in reference No. [26]).

According to a survey by the Japanese Association of Correctional Medicine, 116 (2.7%) of 4,240 prison inmates throughout the Japanese women’s prison system were diagnosed with eating disorders as of October 1, 2011 [25]. The prison sentences for these patients resulted from a variety of crimes; the most frequent of which was repeated shoplifting (72%). Drug-related offences were the second most common (14%), while other crimes, such as arson, murder, fraud, causing injury, and traffic violations accounted for the remaining 14% [25]. The number of female offenders with eating disorders at correctional facilities in Japan is growing each year. While these numbers may only represent a small sample of the prison population, women’s prisons have experienced significant difficulties in the management of patients with eating disorders who have body-critical complications arising from low body weight in addition to behavioral problems [23,26]. A female inmate diagnosed with an eating disorder is sent to a general prison from a detention center if she is not thought to be in an emergency state, and those with severe eating disorders are usually placed in a sickroom and not expected to undertake prison labor [26]. Each general prison is assigned doctors and nurses and is supplied with a variety of nutritional supplements [25] (Figure 1). Prisons in Japan experience problems such as hunger strikes by inmates, and female prisoners are reported to be susceptible to eating behavior disorders because of their feelings of anger and the constraints of the prison environment [27]. Because many patients with eating disorders continue to lose weight through vomiting and/or anorexia, they fall into a psychosomatic body crisis [25,26], These inmates must be observed vigilantly. If a patient needs treatment for their severe psychosomatic state in a hospital outside prison, two prison officers will be required to stay with the patient, and the medical cost becomes enormous. Thus, many patients with eating disorders in an extremely severe physical condition are transferred to Hachioji Medical Prison, which is the largest national specialty hospital operated by Japan’s Ministry of Justice [26]. Inevitably, patients with eating disorders transferred to Hachioji Medical Prison are limited to those with anorexia nervosa accompanied by body crisis [25,26,28]. Because inmates cannot overeat within the disciplined life of correctional facilities, the health of patients with bulimia nervosa or binge-eating disorder usually does not become severe [26]. Thus, the group of patients with eating disorders that are presented in the current study is not a representative sample of the entire cohort of patients with eating disorders in all of the general prisons in Japan.

This review is presented as a case report describing a specific characteristic in a specific cohort of patients with eating disorders who had been incarcerated in Hachioji Medical Prison for shoplifting. Although this study has many overlaps with the content of our previous study in *BMC Psychiatry* [29], the purpose is to describe more features by citing other findings from the relevant literature.

Additionally, while occasionally there are some male patients with eating disorders in men’s general and medical prisons, very few male patients had eating disorders at the time of incarceration and most of their symptoms were observed as being transient or willful behavioral problems that occurred in response to the special environment [26]. Therefore, in the current study, we do not describe male patients with eating disorders.

### Characteristics of patients with eating disorders in Hachioji Medical Prison

In the three years from 1998, Hachioji Medical Prison treated only four patients with eating disorders. Since then, the number of patients with eating disorders has increased rapidly [25,26,28], and the treatment duration for these patients has been prolonged significantly compared with the previous patients [26,28]. Medical prisons are mandated to manage the personal psychosomatic safety of all inmates, regardless of the wishes for treatment by the individual patient [26]. Refusal to accept treatment and relatively high dropout rates pose a major problem for research on the treatment of anorexia nervosa in general hospitals [30]. However, even if a patient refuses treatment in a penal institution, discontinuation of treatment is not an option in a medical prison, because prison administrators have the responsibility of protecting patients from psychosomatic risks [23,26,28,29].

Among the 131 female patients transferred between 2002 and 2011, 70 had an eating disorder; the largest number was in Hachioji medical prison [29]. Among the 67 patients aged under 62 years in this group, the most common convictions resulting in imprisonment were shoplifting (n = 44, 63%) and drug offences (n = 17, 24%) [29].

Here, we first describe the characteristics of patients with eating disorders who were incarcerated for shoplifting and then address patients with eating disorders who had drug problems.

### Patients with eating disorders who were incarcerated for shoplifting (the Shoplifting group)

In most cases, patients in the shoplifting group tend not to have antisocial behaviors and show low frequencies of substance abuse and dangerous behaviors [29]. The Shoplifting group tends to have a significantly higher education history (9–16 years; mean age 13.1 years) than does patients with drug offences (the Drug group) (9–12 years; mean age 9.6 years) [29]. Our earlier study reported that five patients in the Shoplifting group (n = 32) graduated from university and that 11 graduated from professional school [26]. However, the Shoplifting group tends to have prolonged eating disorder histories and maintains a low body weight [23,26]. There were significantly more patients with comorbid obsessive-compulsive disorder in the Shoplifting group (13, 32%) than in the Drug group (0%) [29].

It is often thought that the shoplifting of food by patients with eating disorders arises from a relative shortage of food because food is consumed on a daily basis [25,26]. However, patients in the Shoplifting group included many with restricting type anorexia nervosa (29%) [29]; these patients often had built up a large hoard of food at home [25,26]. Patients with eating disorders who were repeat shoplifters were reported to have significantly higher levels of psychiatric disturbance, depressive illness, and hoarding behavior [31].

Patients with eating disorders most commonly targeted food when shoplifting. Most patients were not impoverished (10%) and had sufficient money to buy food at the time of shoplifting, but responded that they had strong hesitation to spending their money [26]. They had a clear purpose for those foods they stole, that is, they usually wanted to use them for eating or hoarding. We routinely record in the prison record whether or not the patients with eating disorders who were incarcerated for shoplifting meet the Diagnostic and Statistical Manual of Mental Disorders IV-TR diagnosis criteria for kleptomania [26]. Only one patient in Hachioji Medical Prison had a diagnosis of kleptomania [26,29].

Generally, no history of systematic disturbance of consciousness was present at the time of the shoplifting, and they usually apologized promptly at each time of arrest. [23,26]. As for patients in Hachioji Medical Prison, most with eating disorders who were incarcerated for shoplifting only were usually lacking in awareness of the crisis of their disease and incarceration [23,26]. Only 25% of patients in the Shoplifting or Drug groups had been undergoing treatment before their latest arrest [26]. Even after opportunities for exemption from prosecution, the patients in Hachioji Medical Prison had not received treatment or stopped treatment halfway without improvement [26]. It is difficult for patients to consult an expert doctor accustomed to working the with the offenses of patients with eating disorders without a lawyer’s recommendation prior to the patient’s court appearance [26,28]. Many patients with eating disorders in the medical prison ward had been unable to cease shoplifting and were sentenced to prison after two or more suspended sentences [23,26].These patients reported that they did not actually think that they could be incarcerated for their offense, even in a detention center [26]. Most also continued their binge-purging behavior with food from family and/or prison caterers before their actual imprisonment [23]. However, after a conviction was established, the patients tended to show difficulty eating and lost weight rapidly [26]. It is possible that this was influenced by the strict administrative environment of prison, a sense of hopelessness, and a reduction in the amount of available food.

### Patients with eating disorders who were incarcerated for drug-offenses (the Drug group)

Past papers that reported on the impulsivity of patients with eating disorders found that patients with eating disorders who were incarcerated for drug offences (the Drug group) had various histories of substance abuse, impulsive behavioral problems, and child abuse [29]. Previous prosecution and/or incarceration for crimes other than shoplifting was also evident in many patients in the Drug group; many had experience working as prostitutes or night club hostesses (57%), few had experience of full-time work, and many also showed antisocial behaviors (79%) [29].

**Figure 2:**
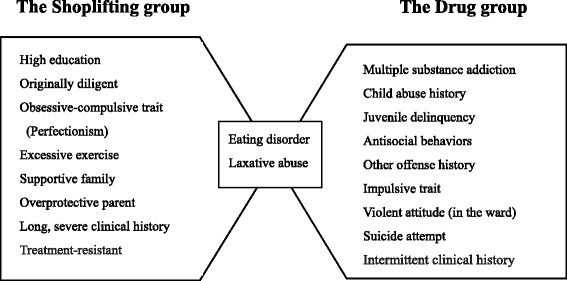
Key words that indicate the characteristics of two groups of patients with eating disorders in Japanese medical prisons. (Reorganized from references [25,26,29]).

There is little overlap in the pattern of behavioral problems between the two groups (Figure 2).

## Treatment in Hachioji Medical Prison

### Management of physical crisis

In this facility, many patients with eating disorders initially refuse to engage in treatment, and even within the well-disciplined prison environment with education about the risk to their bodies, weight-loss actions persist [26,28]. Patients tend to show clinical abnormalities, such as disturbance of consciousness, hypothermia, and electrolyte abnormality. Some also enter a state of shock from pneumonia and pyelonephritis [25].

A psychiatrist provides consistent patient support from the time of their physical crisis through to treatment acceptance in Hachioji Medical Prison [26]. Support from internal medicine doctors, surgeons, and other doctors can be obtained at any time, particularly because the increase in calories during therapeutic feeding must be carefully considered [26,28]. However, to prevent irreversible brain damage, the staff will order compulsory treatment, such as a central venous nutrition catheter, which requires body restraints because some patients repeatedly pull out intranasal nutrition tubes and others intentionally vomit their liquid nutrition [25,26,28].

### Behavioral treatment based custody and other supportive therapy

After a patient in the Shoplifting group recovers from a body crisis, treatment progresses with behavioral therapy, which may be combined with cognitive behavioral and group therapies [26,28]. Any therapy-based restrictions on patients in the ward are lifted when their symptoms and weight improve [26]. It is a violation of prison policy for a patient to have food in their room after mealtime. However, some patients conceal food or vomitus in drawers, beds, washbasins or other hiding places [25,26]. Because the rooms of individual patients in the medical prison are locked and prison officers patrol every 15 minutes, 24 hours a day, behavioral problems are more likely to be detected than in general hospital wards [25,26]. Furthermore, a number of measures have been put in place to allow more accurate observation during treatment, such as inspection of belongings (toilet seats, garbage boxes, bedding, and clothes), restriction of faucets and drain outlets (water usage not allowed without observation by staff), and food given in a separate room with 2-hour postprandial monitoring [25,26].

When the patients are anxious and fearful in the beginning of treatment, we sometimes prescribe antipsychotic drugs such as olanzapine. Since the patients with eating disorder in our ward have strong anxiety in the process of weight gain, we often administer antidepressants that have an anxiolytic effect, such as paroxetine [25,26].

Psychiatrists conduct group therapy with a focus on patients in the Shoplifting group. Because there are always approximately five patients in the Shoplifting group in Hachioji Medical Prison, it is possible to have a weekly group therapy. This therapy is thought to be effective in helping facilitate treatment [25,26]. Patients who initially reject therapy and insist on denying their own illness during group therapy begin to recognize the danger they face and the purpose of therapy in casual conversations with fellow patients who have been in the medical prison longer [25,26]. After a patient attends several months of the group therapy, they can often be seen earnestly admonishing other patients with negative attitudes. Our previous study found that all patients sat in on sessions to observe the group therapy, and all patients, including those expected to object to or be fearful of group therapy, expressed their desire to participate in the next group session [26].

### Behavioral problems during the clinical course

While food refusal and purging behaviors are common in both the Shoplifting and Drug groups during hospital care and treatment, falsifying dietary intake amounts, food hoarding, and excessive exercise are observed more frequently in the Shoplifting group [29]. Rumination is observed in a large number of the Shoplifting group [26], and excessive cleaning, fear of being unclean, and compulsive writing are also prominent behaviors in this group [25,26,28]. It is possible that these behaviors express the obsessive and compulsive characteristics of patients in the Shoplifting group. It is noteworthy that a significantly higher number of patients in the Shoplifting group exercise more excessively (19/42, 46%) than those in the Drug group; they continue to exercise relentlessly and this habit is difficult to suppress [29]. It is possible that their shoplifting occurrs incidentally during a state of confusion, such as from hypoglycemia. Most patients in the Shoplifting group had fallen into a confused state at the time of admission (79%), and they tended to continue their problem behavior [25,26]. However, because their excessive exercise behavior remained even after body-weight recovery, it can be assumed that behaviors such as the recurrence of compulsive shoplifting do not occur solely because of the transient or acute decline of body weight [23,28,29]. Frequent laxative requests are observed in both eating disorder groups [26], although patients in the Shoplifting group tend not to request drugs such as sleeping medications or tranquilizers [26,29]. Many patients in the Shoplifting group (78%) strongly protest against clinical advice from the medical staff during the course of treatment [26], but do not resort to disruptive, violent, or dangerous behaviors such as ranting, making noise, or self-injury [29]. In addition, many patients in the Shoplifting group eventually understand that treatment is necessary to prevent reoffending [25,26].

### Clinical outcome

Although BMI at the time of admission was 11.2 kg/m^2^ in the Shoplifting group and 12.1 kg/m^2^ in the Drug group, the BMI of both at the end of the treatment improved significantly (P < 0.001: 16.4 kg/m^2^, 16.5 kg/m^2^; Shoplifting group, Drug group; respectively) [26]. We do not think this is because we have an especially good treatment method; rather, we think the specific circumstances of treatment in the medical prison that mean it cannot be finished before gaining stable improvement was the major contributor to their recovery. The treatment duration for patients in the Shoplifting group (mean 183 days) was significantly longer than for those in the Drug group (mean 127 days); this may be related to the depth of psychopathology in eating disorders [26]. Although adherence to eating disorder treatment was poor among patients in the Shoplifting group, low body weight and behavioral problems did improve following treatment, without severe sequelae or patient death [26,28]. Some patients complained about the eating disorder treatment in Hachioji Medical Prison, and one patient said she would litigate [25,26]. No previous objection to treatment had been made by families or lawyers, as generally the families of patients in Hachioji Medical Prison are cooperative and are appreciative of treatment [25,26].

Patients with eating disorders are returned to general prisons when they maintain their BMI above 16.0 for 4 weeks and when they no longer exhibit behavioral problems related to their eating disorder other than excessive exercise [26]. Patients are usually transferred to a general prison that is located close to their place of residence, with potential opportunity for parole [26].

### Treatment limitations and future focus

Accommodation of patients in medical prisons has space limitations, and those patients with a physical crisis are prioritized [26,29]. Therefore, the majority of patients in the present study were patients in an anorexic state [25,28,29].

While ideally incarceration of patients with severe disease should be avoided, it may benefit patients who have a severe, prolonged eating disorder and who have either not had treatment or dropped out of treatment, because the patients can experience a return to health owing to a stable treatment process. The unique custodial environment in Hachioji Medical Prison enables the treatment team to treat patients with severe eating disorders that otherwise may not have received help. Outside of an environment like this, eating disorder practitioners are often too busy to take new patients, and the medical setting is often far from the patient’s home.

Following their release from prison, strong support for the patient and regular outpatient treatment is desirable; however, there is potential for a human rights issue to emerge if this is forced upon patients [25,26]. Some eating disorder experts have defended patients prosecuted for shoplifting in court, asserting that treatment should take priority over punishment [23,28]. However, if the patient is re-arrested, probation is revoked and the offender’s imprisonment will become longer [26]. The most important thing is not only to help the patient escape from the impending incarceration, but also to consider the patient’s personal and social circumstances from a long-term perspective.

As general prisons develop experience in the management of eating disorders, their ability to respond to patients improves [28,31]. The Japanese Ministry of Justice has now established a ward providing specialized care for female patients with eating disorders in the Kita-Kyushu medical prison, with some patients already receiving expert treatment [31]. The Correction Bureau within the Ministry of Justice is also considering future measures, with guidance from the Japan Society of Eating Disorders [31].

Study of the neurocircuits involved in planning and interpreting consequences suggest that individuals who have recovered from anorexia nervosa may have difficulty in differentiating positive and negative feedback [32]. It has also been reported that shoplifting is caused by periods of worsened self-esteem, depression, or purging, and that no significant difference exists in long-term criminal tendencies of patients with eating disorders and other patients [33]. Shoplifting by patients with eating disorders is often considered in relation to severe psychopathology [10,18-21].

## Conclusion

It can be said that patients with anorexia nervosa incarcerated for shoplifting are a peculiar group who do not display antisocial characteristics or impulsivity. The psychopathology of their severe eating disorders is probably related to obsessive-compulsive behaviors. It can be assumed that repeated shoplifting behavior may be done by a patient who does not have other criminal or antisocial behaviors if the patient’s eating disorder has been severe and prolonged without treatment. If the mainstream medical system were to place greater emphasis on early-stage protective medical treatment, the number of patients in the care of the justice system including prison would probably decrease. It is difficult for the Japanese judicial authority to manage repeated shoplifting by patients with eating disorders; we think there is the possibility that similar difficulties arise within various judicial institutions worldwide. Further research using a multi-disciplinary approach will be required to reveal how shoplifting relates to eating disorders.
